# Patients Covertly Recording Clinical Encounters: Threat or Opportunity? A Qualitative Analysis of Online Texts

**DOI:** 10.1371/journal.pone.0125824

**Published:** 2015-05-01

**Authors:** Maka Tsulukidze, Stuart W. Grande, Rachel Thompson, Kenneth Rudd, Glyn Elwyn

**Affiliations:** 1 The Dartmouth Center for Health Care Delivery Science, Hanover, New Hampshire, United States of America; 2 The Dartmouth Institute for Health Policy and Clinical Practice, Lebanon, New Hampshire, United States of America; 3 The Scientific Institute for Quality Improvement, Radboud University Medical Center, Nijmegen, The Netherlands; US Army Engineer Research and Development Center, UNITED STATES

## Abstract

**Background:**

The phenomenon of patients covertly recording clinical encounters has generated controversial media reports. This study aims to examine the phenomenon and analyze the underlying issues.

**Methods and Findings:**

We conducted a qualitative analysis of online posts, articles, blogs, and forums (texts) discussing patients covertly recording clinical encounters. Using *Google* and *Google Blog* search engines, we identified and analyzed 62 eligible texts published in multiple countries between 2006 and 2013. Thematic analysis revealed four key themes: 1) a new behavior that elicits strong reactions, both positive and negative, 2) an erosion of trust, 3) shifting patient-clinician roles and relationships, and 4) the existence of confused and conflicting responses. When patients covertly record clinical encounters – a behavior made possible by various digital recording technologies – strong reactions are evoked among a range of stakeholders. The behavior represents one consequence of an erosion of trust between patients and clinicians, and when discovered, leads to further deterioration of trust. Confused and conflicting responses to the phenomenon by patients and clinicians highlight the need for policy guidance.

**Conclusions:**

This study describes strong reactions, both positive and negative, to the phenomenon of patients covertly recording clinical encounters. The availability of smartphones capable of digital recording, and shifting attitudes to patient-clinician relationships, seems to have led to this behavior, mostly viewed as a threat by clinicians but as a welcome and helpful innovation by some patients, possibly indicating a perception of subordination and a lack of empowerment. Further examination of this tension and its implications is needed.

## Introduction

What might happen if patients were to use digital devices such as smartphones to covertly record clinical encounters? Would this behavior constitute a challenge to the social contract between patient and physician, where clinicians perceive their professional autonomy and privacy to be compromised, and respond by denying care to patients who want a digital record of a clinical encounter? Or would this behavior be reasonable, justifiable, and even desirable in light of the widespread goal of more activated and engaged patients? Increasing reports of the practice of patient’s covertly recording clinical encounters suggests that these are no longer hypothetical questions[1][2].

It seems that the growing emphasis on patient engagement [3] [4], health activism and consumerism [5], coupled with the ubiquity of recording devices, has given rise to a new trend of patients recording clinical encounters, with some acting covertly. This phenomenon has generated controversial reports in the media over the last few years (e.g., Reuters [2], National Post [1]). Yet, the topic has not been well researched; we could find only two articles in the peer-reviewed literature. Twenty-five years ago, Goldstein [6] reported two cases of surreptitious surveillance of forensic psychiatric examination by patients, and discussed moral arguments for and against the practice. Goldstein’s prediction that “this may become an increasingly familiar phenomenon” was prescient, if premature. The second, an opinion piece published in 2012 [7], discussed the legality of secret recording in the context of protected information and its admissibility in legal proceedings, but did not explore the issue further.

In contrast, research extending over 40 years has examined the benefits, risks, and implications of clinicians providing copies of audio-recordings to patients. A recent review that included 33 studies showed that patients reported many benefits of receiving these recordings, including better information recall and understanding [8]. However, despite evidence that patients consistently place a high value on receiving audio-recordings of clinical encounters, the practice is not yet part of routine clinical care. Whether this is a factor in the trend to covert recordings has not been investigated.

The topic of covert recording is therefore in need of further exploration [9]. Our study aim was to examine the phenomenon of patients covertly recording clinical encounters and to analyze the underlying issues. We set out to do this by undertaking a qualitative analysis of Internet-based articles and postings on the topic.

## Materials and Methods

We conducted a literature search for and qualitative assessment of online texts discussing patients covertly recording clinical encounters between 2006 and 2013. Online data was collected over a two- month period in 2013 and qualitatively analyzed by a multidisciplinary team of researchers using a five- step process (S1 Table).

### Data Collection

The data comprised online, English-language posts, articles, blogs, and forums, hereafter referred to as texts, which discussed patients covertly recording clinical encounters. We excluded texts discussing: 1) recording for research or clinical purposes, 2) new technology to improve communication with patients, 3) recording by clinicians (overt or covert), and 4) covert recording where patient and clinician were engaged in both professional and personal (e.g. romantic and/or sexual) relationships. We did not analyze videos or audio postings.

After formulating and piloting six search strings using different keywords (e.g. secret OR covert OR surreptitious patient recording), two researchers (MT, RT) independently conducted searches in *Google* and *Google Blogs*, the most widely used search engines, during September and October 2013[10]. Both researchers independently screened the first 100 search results for each search string. We considered this to be sufficient to achieve data saturation and reviewed potentially relevant texts in full. Search results were compared as a team and final inclusion was based on relevance and overlap. Any ambiguities about inclusion were resolved by consensus in the research team. The complete search strategy is available (S2 Table) and archived search results are also available upon request.

The Dartmouth College Institutional Review Board (IRB) determined that the research project was exempt from further ethical review and, as the source material was publicly accessible, informed consent from contributors was not required.

### Data Analysis

We conducted a thematic analysis to identify patterned meaning across the data [11]. A process of data familiarization, data coding, theme development, and revision allowed us to identify, code, and describe general themes in the data, using an inductive, iterative method.

Each researcher independently examined and coded a subset of the texts identified. Selection was based on scope, breadth, and relevance (T30, T36, T48; S3 Table). The research team discussed, refined, and grouped provisional codes into categories and created an agreed codebook. Next, each researcher independently applied the codebook to a selection of the texts assigned to them, wrote a summary of the content, and proposed themes. The final set of themes and their relationships was developed iteratively based on group discussions. A detailed description of each step in the thematic analysis is provided as well as the final data set.

## Results

Sixty-two texts published in multiple countries between 2006 and 2013 were included (S3 Table). These comprised 12 forums (or discussion boards), 22 news or other articles (online magazine, commentary, or report), 23 blog posts, texts describing two videos and one radio episode and two posts on generic question-and-answer pages. Some texts were accompanied by comment threads with the number of comments ranging from zero to 606 comments.

Thematic analysis revealed four key themes: 1) a new behavior that elicits strong reactions, both positive and negative, 2) an erosion of trust, 3) shifting patient-clinician roles and relationships, and 4) the existence of confused and conflicting responses. Below, we describe these themes in more detail and provide illustrative quotations from the texts.

### Theme 1: A new behavior that elicits strong reactions, both positive and negative

The practice of patients covertly recording clinical encounters was viewed as a new and noteworthy behavior. Reactions to the issue were strong and mixed. Some contributors viewed covert recording as an inevitable development, an *“unstoppable patient initiative*, *well in progress” (Contributor 1*, *T3*), enabled by technology.

*The advent of small*, *powerful*, *relatively cheap*, *and thus now more or less ubiquitous electronic gadgetry means that patients WILL record*, *if they want to*, *and I have direct knowledge of an increasing number who do*. *(Contributor 2*, *lawyer*, *T43)*

The perceived inevitability of recording was the basis for a prominent view in the texts examined that health care clinicians should “*accept the prospect of covert recording as a product of the digital age and ensure that it does not work against you [clinicians]*” *(Contributor 3*, *editor*, *T40)*. This sense of inevitability prompted some contributors to suggest that clinicians should “… *er*, *grow up and get used to it*” *(Contributor 4*, *T48)* and that attempts to prevent the recording would be “*way behind the times*, *and to the disadvantage of both medical practitioners and their patients*” *(Contributor 2*, *lawyer*, *T43)*.

Some texts, mostly contributed by patients and patient advocates, contained references to the benefits of covert recording. For instance, recording was thought to potentially increase patient adherence, help improve understanding of clinical information, and improve the quality of care. For many patients covert recording was also justified as a means to obtain verifiable evidence of potentially poor quality care, including data that could be possibly used to support a complaint. Some patients also felt recording would empower them against “*doctors [who] rely on our ignorance…and retreat quickly when faced with an informed woman*…*” (Contributor 5*, *patient*, *T23)*.

Others who were positive about the value of covert recordings identified themselves as lawyers, bioethicists, or patient advocates. They viewed recordings as “*a legitimate tool to keep the healthcare system open and honest about its failings*, *and it should not be prohibited or punished*” *(Contributor 6*, *editor*, *T37)* and felt patients should be encouraged to record their clinical encounters. Moreover, they felt that recordings might provide protection for patients “*who are unhappy with their care*” *(Contributor 6*, *editor*, *T37)*.

Some clinicians also noted possible benefits of recording, for instance, the awareness of being recorded might act as “*an incentive to be at the top of my game*” *(Contributor 7*, *physician*, *T30)* or as protection against litigation. However, the texts mostly contained clinicians’ strong negative reactions, such as the following extract that adopted sarcasm to highlight the perceived inappropriateness of the practice.

*OK*, *I am a physician*. *I guess I just as well might videotape every exam of every patient whether they want to or not*. *This way what I do and how I do it will also be recorded*, *and the patients’ cooperation or lack there of [sic] will also be recorded*. *This will help me avoid frivolous lawsuits*. *Everyone on board*? *(Contributor 8*, *physician*, *T48)*

Clinicians who expressed that recordings can be valuable and helpful for patients as a means to aid recall of complex information were also unhappy with covert recording, and indicated a preference for patients to ask for permission.

Many clinicians viewed covert recording as a violation of their professional rights and privacy, as an invasion of a space deemed by them to be under their authority, and as an impediment to open conversation. Most expressed fears that covert recording would damage the therapeutic alliance and potentially drive a defensive approach to medical practice. They also expressed concerns that the recordings would be misused, tampered with, or made public. Clinicians who described discovering that patients had covertly recorded a clinical encounter also described their negative emotional responses including outrage, anger, surprise, and embarrassment. A number of clinicians described confiscating the recording devices or dismissing the patients from their practice.

Primarily, writing in defense of the practice of covert recording, patients questioned the notion that clinicians should be granted special protection from recording. They also questioned physicians’ “*superiority*” and “*elevated*” standing in society and called for greater physician accountability.

*Why should doctors be insulated from the checks and balances—that the rest of us are subject to*?… *This is another example of how we coddle doctors in this society*. *(Contributor 9*, *T48)*

However, other patients countered these sentiments by emphasizing the complexity of clinical mastery of clinicians who are *“*… *almost all doing their best to serve” (Contributor 10*, *patient*, *T48)*.

*Doctors are not mechanics*, *or accountants*. *How can we imply that they should be held liable [sic] as such*? *(Contributor 11*, *patient*, *T48)*

Both patient and clinician responses reflected a sense of unfair treatment and double standards. Patients perceived that clinicians were permitted to record visits as needed while they were not afforded the same right. Clinicians cited strict requirements to obtain patients’ consent for any recording while the same “*is not true when a patient wishes to make a recording of a consultation*” *(Contributor 3*, *editor*, *T40)*.

### Theme 2: An erosion of trust

A prominent view in the majority of texts was that covertly recording clinical encounters by patients signaled a loss or absence of trust in the health care system. Some justified covert recording because of a belief that the care process would not meet their expectations, while others cited previous experiences of poor care and inadequate responses to their concerns as justifications.

*Obviously bringing concerns to the authorities doesn't work*. *No one is ever held accountable*, *no proof yada yada*. *(Contributor 12*, *T36)*

At times, the apparent erosion of trust in the health care system was extreme, with some contributors expressing very negative views of clinicians when justifying covert recording.

*…ALWAYS record EVERYTHING*. *These people [physicians] can lie*, *cheat and steal and act immorally*…*and do so regularly*. *(Contributor 13*, *T36)*

Moreover, it appeared that patients’ lack of trust that clinicians would support of *overt* recording, combined with the perceived value of having a recording of the encounter, was a driver of their decision to act covertly.

*It's only in the absence of that knowledge [permission to record] that I'm acting surreptitiously -­-­ and would continue doing so were I to find out I did NOT have the right to record*, *since I'd find more value in having the recordings than in following that law*. *(Contributor 14*, *patient*, *T31)*

As well as representing a consequence of eroded trust, covert recording was also seen as a contributor to a further deterioration of trust between patients and clinicians. This view was strongly held by clinicians who saw covert recording as an illegitimate and “*trust-busting*” behavior.

*Any covert recording would seem inherently intrusive and a breach of trust in a patient-dentist relationship*. *(Contributor 15*, *dental adviser*, *T41)*

Clinicians were especially alert to the legal threat posed by recording clinical encounters. As one clinician recounted, “*She [patient] said her lawyer told her to tape every visit*, *[because] sooner or later I’d make a mistake and then she could make lots of money” (Contributor 16*, *physician*, *T48)*.

In addition to attributing to a fear of being *prohibited* from recording or admonished, some contributors suggested that the practice was a last resort for patients who felt they had exhausted other channels for meeting their goals. This distrust in both physicians and the health care system was accompanied by a belief that the practice might be overcome through open and honest discussions “*in order to avoid them feeling it necessary to go ‘undercover’” (Contributor 15*, *dental adviser*, *T42)*.

Notably, the issues around trust expressed by some patient contributors were also raised by others, including members of the legal profession.

*I have been consulted by several patients who wish to record all medical appointments—and now do—and would like to do so openly*. *However*, *[sic] I advise them to do so on an undeclared basis*, *precisely because medical practitioners\hospital administrators routinely try to bully such patients into not recording (by delaying their consultations\treatment while 'policy' is considered*, *or by threatening to remove them from GP practice lists*, *for instance)*. *(Contributor 2*, *lawyer*, *T43)*

### Theme 3: Shifting patient-clinician roles and relationships

Discussions about the phenomenon of covert recording emphasized perceived shifts in the dynamics of health care delivery; particularly, in relation to power, control, and ‘ownership’ of the clinical encounter.

Advocates of covert recording often viewed the behavior as an effective and legitimate way for patients to acquire or assert more power in the health care process than has previously been afforded to them, indicating possible perceptions of subordination and lack of empowerment. Clinicians’ negative reactions were similarly seen as evidence of their unease with this shift:

*I think there is an underlying ‘dynamic’ in the perceived problem*: *it represents patients’ taking an initiative as regards to [sic] their own healthcare which doctors cannot ‘control’*, *and that just doesn’t suit some doctors’ sense of self-importance*. *(Contributor 4*, *T48)*

Comments that referred to the clinical encounter as a space requiring their control seemed to confirm this view.

*Doctors will need to […] protect themselves and to protect the privacy of the patient-physician encounter*. *(Contributor 17*, *Physician*, *T7)*

Debates of the ethical or legal appropriateness of recording clinical encounters often centered on which party had greater ‘ownership’ of the encounter, the information exchanged, and any enduring record of that exchange.

*Wouldn't a conversation that is recorded in the doctor's office technically be considered part of a medical record*, *aka protected health information*, *subject to the same rules and regulations as HIPAA*? *That conversation technically belongs to the doctor… (Contributor 18*, *T30)*

*When a patient seeks a consultation […]*, *the information being processed is almost exclusively relating to the patient*. *Under the Data Protection Act*, *that data is therefore personal to the patient*. *By recording it*, *that patient is merely viewed as processing their own data*. *(Contributor 15*, *dental adviser*, *T42)*

Clinicians’ views that covert recording is a violation of their rights and privacy also appeared strongly related to issues of control and power. As one clinician put it, *“Why would a patient or family want to record our actions*? *Are they upset about our care or waiting to catch proof of a mistake*? (Contributor 19, T15). This discordance in perspectives reflected disagreement between clinicians and patients as to the social contract that underpins the relationship. Despite resistance from clinicians, patients expected full transparency and equal ownership:

*These people [clinicians] are NOT in positions of authority*, *we contract their services and ask for their advice based on their expertise and training*, *and we are 100% free to tell them we think their advice is sub-standard*…*(Contributor 20*, *patient*, *T17)*

Describing the health system as ‘broken’, patients said their attempts to seek redress through formal channels had been unsatisfactory. Many felt it was a futile task to complain; their concerns carried no weight against the response of the system. In contrast, critics of covert recording noted the availability of existing processes and saw the behavior as indefensible.

### Theme 4: The existence of confused and conflicting responses

In the texts, both patients and clinicians sought legal counsel and guidance about whether covertly recording clinical encounters was legal or ethical, often generating long threads of comments and discussions.

*Can I record a conversation with my doctor with out his consent*? *(Contributor 21*, *patient*, *T29)*,

*Would any of the practicing physicians here remove a patient from their care if you found out your patient was secretly recording you*? *(Contributor 22*, *physician*, *T30)*

Many individuals called for action to clarify or implement rules and regulations, with some offering suggestions for policies.

*Post a sign … ‘Any type of electronic recording is strictly prohibited at any location within these offices*.*’ (Contributor 23*, *malpractice insurer*, *T52)*

*The doctor and patient mutually agree that they will not use video or audio recording equipment without express written permission of the other party*. *(Contributor 24*, *T30)*

Policy recommendations ranged from “*no recording*” to an open recording policy. Some contributors viewed *“videotaping a doctor [as] a form of electronic note-taking that doesn’t require consent” (Contributor 6*, *editor*, *T37)* or, indeed, any regulation.

The texts contained evidence of variation in regulatory responses to covert recording across countries, which seemed related to different degrees of ‘system readiness’. In the United States, for example, there seemed to be less acceptance of covert recording than in the United Kingdom, related to differences in the legality of the practice.

Admissibility of covert recordings as evidence in legal settings was also discussed at length. Discussions related to concerns of privacy and confidentiality, both for the patient and clinician, but no reports or ways to overcome these worries were given.

## Discussion

The four key themes identified in this study indicate that the covert recording of clinical encounters by patients is a new behavior, enabled by digital device ownership. The behavior elicits strong negative reactions, particularly from clinicians. It was seen as a consequence of distrust in the health care system and, when identified, a cause of further erosion in trust. Covert recording was viewed as part of an ongoing shift in patients’ and clinicians’ roles and relationships in medicine. Confused and conflicting responses to the phenomenon by patients and clinicians highlight the need for policies and legislative guidance.

This study had several strengths. We applied thematic analysis to assess texts from a wide range of online contributors (patients, physicians, dental advisors, advocates, lawyers, and insurers) to explore naturally occurring reactions, unaffected by observation. Exploring naturally occurring texts in online platforms allows researchers to examine attitudes that are freely and openly expressed [12]. Such texts lend authenticity to experiences that are hard to capture using more traditional techniques. However, the method has limitations. First, our analysis was limited to the data expressed in texts and as such, we were unable to assess the influence of contributor characteristics or underlying prejudices [13][14]. We are also unable to comment on the representativeness of the views expressed in the analyzed texts. We admit that the views expressed are likely to represent those who are willing to express strong opinions on the topic of covert recording, both positive and negative, than the broader population. We have no data to indicate how prevalent this phenomenon has become. Recognizing that using additional search engines may have yielded more sources, the likelihood of these sources changing the overall findings of our analysis (the four major themes) is highly unlikely.

These limitations are, however, balanced by several strengths. First, our analysis captured reactions to covert recording of clinical encounters over 7 years by a diverse set of respondents. Second, the thematic analysis benefited from the perspectives of a multidisciplinary team, comprising both clinicians and researchers.

The study reveals that the underlying motivations for covert recording by patients are multidimensional and complex. Lack of trust in physicians and health care systems as well as shifting roles and responsibilities [15] has emerged indicating issues of wider societal trust in our study. The recent development of allowing patients more open access to their medical records has stimulated similar discussion[16]. In a similar vain, digital recordings of clinical encounters signal a call for even more transparency about clinical interactions.

## Supporting Information

S1 Table Data analysis and theme generation. (DOCX)

S2 Table Search Strategy. (DOCX)

S3 Table Text identified and analyzed (n = 62). (DOCX)
