# Supplementary material for: Patients Covertly Recording Clinical Encounters: Threat or Opportunity? A Qualitative Analysis of Online Texts
Source: PLoS One. 2015 May 1;10(5):e0125824. doi: 10.1371/journal.pone.0125824 (PMC4416897; doi:10.1371/journal.pone.0125824)
Supplement: S1 Table — (DOCX) [file pone.0125824.s001.docx]

| **Table 1.** Data analysis and theme generation | |
| --- | --- |
| **Data Analysis Steps** | **Description** |
| ***Data exploration*** | Reading the text for overall understanding of scope and context |
| ***Development of an initial codebook*** | All researchers (MT, SG, RT, KR, GE) independently coded three texts (T30, T36, T48, Table 2, Appendix 1) selected from the pool of included texts prior to full analysis to create a preliminary coding structure. Two researchers (MT, SG) discussed these initial codes, which were granular and reflective of the explicit content in the selected texts, and grouped them into categories and sub-categories, which became the codebook. Twenty-nine main code phrases were generated with three of them having sub-codes. Next, they were grouped into 10 major code categories with multiple sub-codes. |
| ***Independent analysis of texts*** | All researchers (MT, SG, RT, KR, GE) coded a set of unique texts assigned to them and entered all codes into a Google-based spreadsheet. The spreadsheet columns were set up to allow the population of the codes, subcodes, text identification number, page, paragraph, researcher comment and quotations from the texts in rows. One new code category was generated and seven additional sub-codes were added in the process of analysis and codebook refinement. Minor wording adjustments and modifications were made to existing codes for clarity. |
| ***Reviewing codes, developing a cluster map of themes, and building consensus around structure of themes and inherent relationships*** | Each researcher summarized the content and general themes in their assigned set of texts. Codes and broader patterns (candidate themes) were discussed by the entire research team to develop an initial map of themes. Two researchers (MT, SG) independently reviewed a sample of texts coded independently by each researcher to ensure consistency in coding reliability. |
| ***Reviewing themes*** | Common themes were organized into meaningful categories, which were mapped to emphasize the key themes and relationships among the data. Following the group assessment, two researchers (MT, SG) reviewed and refined the overall cluster map of themes and subthemes. |
| ***Defining and characterizing themes*** | Based on collective assessment and detailed analysis of each theme, the entire research team discussed, defined and named final themes collaboratively. |
