# Supplementary material for: Patients Covertly Recording Clinical Encounters: Threat or Opportunity? A Qualitative Analysis of Online Texts
Source: PLoS One. 2015 May 1;10(5):e0125824. doi: 10.1371/journal.pone.0125824 (PMC4416897; doi:10.1371/journal.pone.0125824)
Supplement: S2 Table — (DOCX) [file pone.0125824.s002.docx]

**Table 2.** Search strategy

List if search strings with different keywords

1. Secret OR covert OR surreptitious patient recording
2. Secret OR covert OR surreptitious patient doctor recording
3. Patients recording doctor visits
4. Recording clinical consultation
5. Surreptitious recordings
6. Patient secret recording

Search results screened for each string*

| Search string | Secret OR covert OR surreptitious patient recording | Secret OR covert OR surreptitious patient doctor recording | Patients recording doctor visits | Recording clinical consultation | Surreptitious recordings | Patient secret recording |
| --- | --- | --- | --- | --- | --- | --- |
| Included (n=62) | 24 | 10 | 25 | - | 1 | 2 |
| Excluded (n=495) | 73 | 76 | 69 | 97 | 96 | 84 |
| Duplicates (n=43) | - | 15 | 7 | 3 | 4 | 14 |
| **n=100 search results screened for each string* | | | | | | |
