# Supplementary material for: Patients Covertly Recording Clinical Encounters: Threat or Opportunity? A Qualitative Analysis of Online Texts
Source: PLoS One. 2015 May 1;10(5):e0125824. doi: 10.1371/journal.pone.0125824 (PMC4416897; doi:10.1371/journal.pone.0125824)
Supplement: S3 Table — (DOCX) [file pone.0125824.s003.docx]

| **Table 3.** Text identified and analyzed (n=62) | | | | | |
| --- | --- | --- | --- | --- | --- |
| **Text number** | **Title** | **Date of publication, country** | **Number of comments** | **Source** | **Truncated URL** |
|  | **Blogs (n=23)** |  |  |  |  |
| T1 | Recording independent medical examinations and workcover telephone conversations | 2013, Australia | 11 | A Diary of a WorkCover Victim | http://aworkcovervictimsdiary.com |
| T2 | Do doctors have to sign Conscientious Objector forms? | 2012, Australia | 163 | Australian Vaccination Network | http://avn.org.au |
| T3 | Using my smartphone in hospital – but shh, don’t tell (I’m recording) | 2013, Canada | 12 | The Front Door to Healthcare | http://frontdoor2healthcare.wordpress.com |
| T4 | Big Brother is Watching? Secret Recordings of MDs | 2012, Canada | 7 | Dr. Brian Goldman, CBC Radio-Canada | http://www.cbc.ca/whitecoat/blog |
| T5 | Secret patient recordings – are they legal? | 2012, UK | 1 | PHIprivacy | http://www.phiprivacy.net |
| T6 | Man Jailed After Comments Made In Atos Assessment | 2013, UK | 384 | Johnny Void | http://johnnyvoid.wordpress.com/ |
| T7 | If patients want to record visits isn’t this just a symptom of your failure to provide them with documentation? | N/A, UK | 0 | mHealth Insight | http://mhealthinsight.com |
| T8 | Secretly recording conversations with doctors… is it legal? | 2012, US | 0 | Mishkind Law Firm | http://www.mishkindlaw.com/blog |
| T9 | Some patients seek to fight malpractice with video recordings | 2013, US | 0 | Chambers, Aholt & Rickard, LLP | http://www.atlantalinjurylawblog.com |
| T10 | Should patients be allowed to record their office visit? | 2011, US | 12 | KevinMD | http://www.kevinmd.com/blog |
| T11 | Secret Recording Held Admissible in a Medical Malpractice Lawsuit | 2012, US | 0 | Post, Health Law Blog for ER Doctors | http://erdochealthlawblog.blogspot.com |
| T12 | Dr Roni Zeiger, Chief Health Strategist, Google: “you should walk into your doctor’s office with a video camera or tape recorder” | 2010, US | 3 | mHealth Insight | http://mhealthinsight.com |
| T13 | How Smartphones Put Your Practice in Danger | 2013, US | 1 | Power Your Practice | http://www.poweryourpractice.com |
| T14 | Take an Audio Recording of Your Doctor’s Office Visit | 2012, US | 0 | Compass Healthcare Consumerism | http://www.compassphs.com/blog |
| T15 | Don't listen to me! Recording without consent in healthcare | 2012, US | 0 | Pro Responder | http://proresponder.blogspot.com |
| T16 | Doctors on the Record | 2013, US | 8 | 33 charts | http://33charts.com |
| T17 | Indiana Doctor tells Mom: “Be Compliant! Vaccinate or I’ll Call CPS!” | 2012, US | 118 | VaxTruth | http://vaxtruth.org |
| T18 | Why every patient should be recording appointments | 2013, US | 8 | Center for Communication in Medicine | http://www.speaksooner.org |
| T19 | Patients increasingly recording doctors' visits | 2012, US | 0 | Bell Media Radio | http://www.newstalk1010.com |
| T20 | ‘OK Glass, Start Patient Record’ Tech That Changes The Patient Dialog | 2013, N/A | 0 | The Doctor Weighs in (TDWI) | http://www.thedoctorweighsin.com |
| T21 | This encounter is being recorded | 2011, N/A | 2 | HealthBeat | http://healthbeat.areavoices.com |
| T22 | When Patients Audio Record Without Your Consent | 2010, N/A | 3 | RangelMD | http://rangelmd.com |
| T23 | Three Ways to Combat Doctors Bullying You Into Unwanted Pap Tests and Pelvic Exams | 2012, N/A | 48 | forwomenseyesonly | http://forwomenseyesonly.com |
|  | **Posts on Forum/Discussion Board (n=12)** |  |  |  |  |
| T24 | Audio-recording your consultations with NHS doctors | 2010, UK | 606 | The consumer action group | http://www.consumeractiongroup.co.uk |
| T25 | Atos Audio Recording Guidance | 2013, UK | 7 | WhatDoTheyKnow | https://www.whatdotheyknow.com |
| T26 | Any *decent* web sites about implementation of covert recording? | 2006, UK | 11 | hydrogenaudioaudio | http://www.hydrogenaudio.org |
| **Table 2.** Text characteristics (n=62), 2006-2013 (continued) | | | | | |
| T27 | Visits "On the Record" | 2013, UK | 4 | Tribe | http://tribe.soapware.com |
| T28 | Audio recording your consultation with NHS doctors | 2013, US | 29 | Phoenix Rising | http://forums.phoenixrising.me |
| T29 | Recording a Doctor Conversation without Him Knowing in South Carolina | 2010, US | 12 | Workers Compensation Insurance | http://www.workerscompensationinsurance.com |
| T30 | Secretly recording doctors during visits | 2010, US | 35 | The Student Doctor Network | <http://forums.studentdoctor.net/> |
| T31 | Recording my therapy sessions | 2007, US | 18 | SelfhealpMagazine | http://selfhelpmagazine.com |
| T32 | Recorded my doctor at MMI visit | 2009, US | 4 | North Carolina Worker's Compensation | http://www.workerscompensationinsurance.com |
| T33 | Should you secretly tape patient conversations? | 2012, US | 15 | Medscape | http://boards.medscape.com/forums |
| T34 | Recording doctor's visit? | 2011, US | 8 | Cholangiocarcinoma Foundation | http://www.cholangiocarcinoma.org |
| T35 | Court Rules Family CAN Use Secret Recording In Negligence Suit | 2012, N/A | 0 | Nurse Anesthetist (CRNA | http://www.nurse-anesthesia.org |
|  | **Online Articles (n=22)** |  |  |  |  |
| T36 | The latest trend in health care: Patients secretly filming doctors | 2012 Canada | 104 | National Post | http://news.nationalpost.com |
| T37 | Why are more patients starting to secretly film doctors? | 2013, Canada | 0 | Technology for Doctors Online | http://www.canhealth.com |
| T38 | On the record | 2012, New Zealand | 0 | The Association of Salaried Medical  Specialists | <http://www.asms.org.nz> |
| T39 | Recording Consultations | 2012, New Zealand | 0 | Medical Protection Society | http://www.medicalprotection.org |
| T40 | Jim Killgore, Covert patient recording | 2012, UK | 0 | Medical and Dental Defense Union of Scotland | http://www.mddus.com |
| T41 | Are your patients recording you? | 2012, UK | 0 | Dental Republic | http://www.dentalrepublic.co.uk |
| T42 | Covert recording risks for dentists | 2012, UK | 4 | Article ProDentalCPD | http://www.prodentalcpd.com |
| T43 | Secret audio recordings: know the law | 2008, UK | 2 | Health Service Journal | http://www.hsj.co.uk |
| T44 | Dilemma: Recording consultations. A patient asks to record my consultation – What should I do? | 2012, UK | 0 | Medical Protection Society | http://www.medicalprotection.org/uk |
| T45 | Personal Independence Payment audio recording of face-to-face consultations | 2013, UK | 0 | Department of Work and Pensions | http://www.dwp.gov.uk |
| T46 | Smartphones in the office: Are patients recording everything? | 2012, US | 3 | FierceHealthcare | http://www.fiercepracticemanagement.com/story |
| T47 | “I am under a lot of pressure to not diagnose PTSD” | 2009, US | 72 | Salon Media Group | http://www.salon.com |
| T48 | iPhone app makes doctors iRate | 2010, US | 23 | Reuters | http://blogs.reuters.com |
| T49 | Pros and cons of letting patients record doctor visits. What should physicians do if patients want to record their discussions? | 2012, US | 0 | American Medical News | http://www.amednews.com |
| T50 | Smile, You Are On Candid Camera | 2012, US | 0 | Law Office of Deniza Gertsberg | http://www.gertsberg.com |
| T51 | There isn't an app for that: Banning recording in the office | N/A, US | 0 | The doctors company, physician-owned medical malpractice insurer | http://www.facs.org |
| T52 | Discouraging Recording in the Office | 2011, US | 0 | American Academy of Otolaringology - Head and Neck Surgery Bulletin | http://aaobulletin-365.ascendeventmedia.com |
| T53 | Is it Legal to Use a Nanny Cam in a Nursing Home? | N/A, US | 0 | TechMedia | http://nanny-cam-review.toptenreviews.com |
| **Table 2.** Text characteristics (n=62), 2006-2013 (continued) | | | | | |
| T54 | Family may use secret recording in medical negligence suit | 2012, US | 0 | American Medical News | http://www.amednews.com |
| T55 | A Case Report from the Anesthesia Incident Reporting System | 2012, US | 0 | Article, American Society of Anesthesiologists Newsletter | https://www.asahq.org |
| T56 | What to Do During Your Doctor Visit | 2013, N/A | 0 | About.com | http://patients.about.com |
| T57 | State by State Compliance | 2010, N/A | 0 | Vegress | http://www.vegress.com |
|  | **Texts accompanying Videos (n=2)** |  |  |  |  |
| T58 | Doctor calls for secret filming ban after ACA sting | 2013, Australia | 75 | The Sydney Morning Herald | http://www.smh.com.au |
| T59 | Secret recording of patient abuse from Sunderland Doctor | 2011, UK | 58 | YouTube | http://www.youtube.com |
|  | **Question (n=2)** |  |  |  |  |
| T60 | Can I legally record a doctor’s visit if I think he's going to lie on the final reports? | 2010, US | 3 | Avvo, legal Q&A forum and directory | http://www.avvo.com |
| T61 | Frequently Asked Questions | N/A, US | 0 | Verilogue, Inc. CareCoach | [http://www.carecoach.com/faq](https://www.google.com/url?q=http://www.carecoach.com/faq&usd=2&usg=ALhdy28FwtNsTc5VQGFLaHtEyzsxY1YYqg) |
|  | **Texts accompanying Radio Clips (n=1)** |  |  |  |  |
| T62 | Covert recording erodes medical relationships | 2012, Canada | 0 | CBC, Radio-Canada | http://www.cbc.ca |
|  | | | | | |
